# Machine Learning-Assisted Low-Dimensional Electrocatalysts Design for Hydrogen Evolution Reaction

**DOI:** 10.1007/s40820-023-01192-5

**Published:** 2023-10-13

**Authors:** Jin Li, Naiteng Wu, Jian Zhang, Hong-Hui Wu, Kunming Pan, Yingxue Wang, Guilong Liu, Xianming Liu, Zhenpeng Yao, Qiaobao Zhang

**Affiliations:** 1 College of Chemistry and Chemical Engineering, and Henan Key Laboratory of Function-Oriented Porous Materials, Luoyang Normal University, Luoyang, 471934 People’s Republic of China; 2 New Energy Technology Engineering Lab of Jiangsu Province, College of Science, Nanjing University of Posts and Telecommunications (NUPT), Nanjing, 210023 People’s Republic of China; 3 School of Materials Science and Engineering, University of Science and Technology Beijing, Beijing, 100083 People’s Republic of China; 4 Department of Chemistry, University of Nebraska-Lincoln, Lincoln, NE 8588 USA; 5 Henan Key Laboratory of High-Temperature Structural and Functional Materials, National Joint Engineering Research Center for Abrasion Control and Molding of Metal Materials, Henan University of Science and Technology, Luoyang, 471003 People’s Republic of China; 6 National Engineering Laboratory for Risk Perception and Prevention, Beijing, 100041 People’s Republic of China; 7 Center of Hydrogen Science, Shanghai Jiao Tong University, Shanghai, 200000 People’s Republic of China; 8 State Key Laboratory of Metal Matrix Composites, School of Materials Science and Engineering, Shanghai Jiao Tong University, Shanghai, 200000 People’s Republic of China; 9 State Key Laboratory of Physical Chemistry of Solid Surfaces, College of Materials, Xiamen University, Xiamen, 361005 People’s Republic of China

**Keywords:** Machine learning, Hydrogen evolution reaction, Low-dimensional electrocatalyst, Descriptor, Algorithm

## Abstract

Efficient electrocatalysts are crucial for hydrogen generation from electrolyzing water. Nevertheless, the conventional "trial and error" method for producing advanced electrocatalysts is not only cost-ineffective but also time-consuming and labor-intensive. Fortunately, the advancement of machine learning brings new opportunities for electrocatalysts discovery and design. By analyzing experimental and theoretical data, machine learning can effectively predict their hydrogen evolution reaction (HER) performance. This review summarizes recent developments in machine learning for low-dimensional electrocatalysts, including zero-dimension nanoparticles and nanoclusters, one-dimensional nanotubes and nanowires, two-dimensional nanosheets, as well as other electrocatalysts. In particular, the effects of descriptors and algorithms on screening low-dimensional electrocatalysts and investigating their HER performance are highlighted. Finally, the future directions and perspectives for machine learning in electrocatalysis are discussed, emphasizing the potential for machine learning to accelerate electrocatalyst discovery, optimize their performance, and provide new insights into electrocatalytic mechanisms. Overall, this work offers an in-depth understanding of the current state of machine learning in electrocatalysis and its potential for future research.

## Highlights

- The process of machine learning is introduced in detail.
- Recent developments in machine learning for low-dimensional electrocatalysts are briefly reviewed.
- Future directions and perspectives for machine learning in hydrogen evolution reaction are critically discussed.

## Graphical abstract

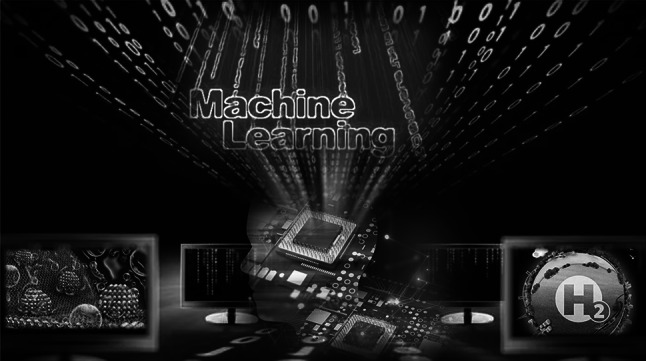

## Introduction

With the urgency of achieving carbon neutrality, green energy sources are becoming more important in promoting economic and social growth [1–5]. Hydrogen, as an ideal renewable energy, has gained great attention [6–8]. Hydrogen generation from the electrolyzed water is the most efficient and sustainable approach to producing high-purity hydrogen [9–14]. However, hydrogen evolution reaction (HER) requires an electrocatalyst with high activity to reduce overpotential substantially. Therefore, more effort is needed in electrocatalyst discovery. Although electrocatalysts play a critical role in the HER, many are still developed through a trial-and-error approach in experimental settings [15–17]. However, given the vast materials space, it is challenging to explore and design exceptional electrocatalysts [18]. Furthermore, the identification of ultrafast catalytic HER atomically is difficult and expensive. Consequently, effective methods for screening electrocatalysts and understanding the HER mechanism are highly needed to be developed.

Low-dimensional materials present a distinctive edge in the context of the HER. Here are some key advantages: (1) High surface-to-volume ratios: these unique geometries endow a multitude of active sites for the HER. The consequent amplification in surface area bolsters catalytic activity, thereby augmenting the efficiency of hydrogen evolution; (2) Enhanced charge transport: low-dimensional materials often exhibit excellent electron mobility, allowing for efficient charge transport. This leads to a decrease in overpotentials, reducing the energy required to drive the reaction and thereby enhancing its efficiency; (3) Tailorable electronic structure: the electronic properties of low-dimensional materials can be finely tuned through chemical modification or by introducing defects. This customization allows for optimized binding energies for hydrogen adsorption and desorption, critical for the HER process; (4) Structural flexibility: the mechanical properties of low-dimensional materials can also be tailored to support optimal structural dynamics. This flexibility can enhance the reaction kinetics, thus making the HER process faster; (5) Integration and synergy: the adaptability of low-dimensional materials allows them to be seamlessly amalgamated with other catalysts or support materials to forge hybrid structures. This engenders synergistic effects, where the ensemble of diverse materials magnifies the overall HER performance. By leveraging these advantages, low-dimensional materials have the potential to significantly improve the efficiency and sustainability of hydrogen production through the HER.

Machine learning has emerged as an important tool for data mining and analysis, and is progressively transforming the way we collect, analyze, and discover materials [19–22], which can aid in the design and discovery of novel low-dimensional electrocatalysts with optimized properties for this important reaction. The increasing popularity of machine learning has revolutionized the predictions of new electrocatalysts, optimal composition, adsorption energy, active sites, electrocatalytic activity, and HER mechanism, enabling researchers to identify these catalysts in a faster and more cost-effective manner compared to traditional experience-based methods [8, 23–26]. For instance, Liu et al. utilized machine learning to investigate the HER process on single-atom catalysts (SACs) doped on 2D GaPS_4_, and illustrated the potential of SACs in catalyzing HER on 2D GaPS_4_ [27]. It provides an effective and economical method to predict HER performance for various low-dimensional catalysts. Wexler et al. adopted machine learning to identify key factors affecting the HER performance of Ni_2_P doped with nonmetal [28]. It was found that the Ni–Ni bond length was the most significant descriptor, indicating that nonmetal doping could enhance reactivity by inducing a chemical pressure effect on the Ni_3_-hollow site. These results provide important insights for the design of new HER electrocatalysts. Moreover, Pandit et al. employed enhanced eXtreme Gradient Boost Regression models to select NiCoCu alloy-based catalysts for the HER, and demonstrated that this approach successfully screened the most active HER catalysts from a vast array of candidates [29]. To investigate structure/property relationships, Parker et al. analyzed 1300 platinum ensembles by machine learning. Their findings revealed that small particles were conducive to disordered materials, while ultra-large (110) surface areas were supportive of ordered materials to achieve efficient hydrogen evolution [30]. Besides, machine learning can be utilized to design experiments for fabricating materials instead of designing materials themselves [31–33]. Despite considerable research on low-dimensional electrocatalysts for the HER with machine learning, a comprehensive summary of this approach is still lacking. Hence, it is crucial to review recent advancements in the machine learning applications to low-dimensional HER electrocatalysts.

In this work, we first present a detailed introduction of the general scheme of machine learning, encompassing data collection, feature engineering, machine learning algorithms, as well as model optimization. Subsequently, we summarize the latest advancements in low-dimensional electrocatalysts guided by machine learning, specifically focusing on their application to the HER. A particular emphasis is placed on comprehending the descriptor performance and enhancing the scope of the application and design effectiveness. Finally, future development prospects and directions for machine learning methods in HER research are proposed.

## General Process of Machine Learning

**Fig. 1:**
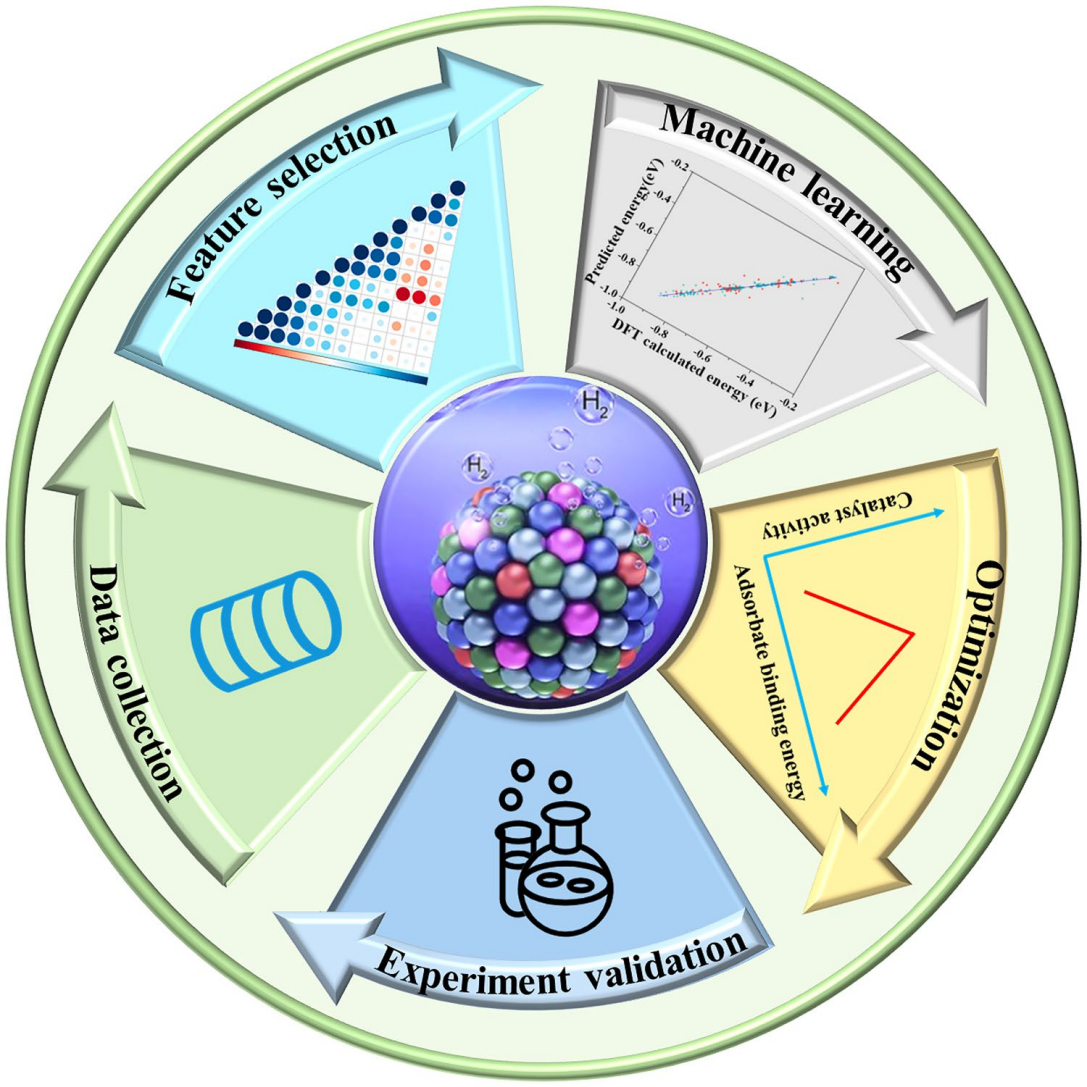
Process of machine learning for designing HER electrocatalysts

When compared to traditional hard-coded screening methods, in which algorithms were prearranged by human specialists prior to their use, computers are capable of learning from training data in the middle of a machine-learning process, allowing them to screen electrocatalytic materials automatically [34–36]. A machine learning model can be constructed as a result of linear or nonlinear relationships that exist between features and material properties, and it is through this fundamental step that the applicability and feasibility of the model can be evaluated [37–39]. Subsequently, using the correct algorithms, models are constructed and predictions are made about reaction mechanisms or properties based on the data presented [40]. Finally, after the training and test sets have been established, the machine learning models are validated, assessed, and optimized [41]. In Fig. 1**,** we illustrate the process of using machine learning-accelerated computations and designing electrocatalysts for the HER. Subsequently, the utilization of machine learning will be described with detailed elaboration.

### Data Collection

In machine learning, the most critical step is the collection of data [42]. To obtain the desired data, a digital format is used, based on the data gathered from the experiment, the calculation, and the simulation, as well as from the database [43]. Furthermore, to integrate data from a variety of sources during the collection process, data fusion is performed [44]. As a result, the merged data is more comprehensive and informative as compared with the original data. Nevertheless, the collected data often contains noise, incorrect, irrelevant, and even missing data, which can significantly compromise the quality of the machine learning model [45]. The quality of the data is evaluated based on various indexes, including validity, accuracy, completeness, consistency, and uniformity [46–49]. Fortunately, databases for materials science have been consistently built over the past century and can provide large raw data, bringing great convenience for machine learning [50–52]. Materials databases, for example, the Inorganic Crystal Structure Database, the Pauling File, the Crystallography Open Database, and similar databases are widely used as data sources [20, 53–55]. Additionally, there is no doubt that the use of databases of computed materials, such as those developed by the Material Project and Open Quantum Materials, is becoming increasingly common [9, 56–59]. Databases for specific applications, including Materials Web online database, Cambridge Structural Database, and Catalyst Acquisition by Data Science, are also adopted [13, 60–64]. Furthermore, low-dimensional materials databases are specialized repositories of information and data related to various low-dimensional materials, such as 2D materials (e.g., graphene, transition metal dichalcogenides) and 1D nanowires (e.g., carbon nanotubes, nanowires). Renowned databases include but are not limited to Materials Project, 2D Materials Database [65], Nanomaterial Registry [66], and Computational 2D Materials Database [67]. These databases serve as valuable resources for researchers and scientists working in the field of materials science and nanotechnology. More importantly, rapid access to materials databases is crucial for data collection in machine learning.

After the data collection process, the collected data may be incomplete, inconsistent, or even spurious, and may not be compatible with the working environment [68]. As a precaution to make sure that predicting models will perform well, irrelevant and duplicate data should be removed. Machine learning models require accurate data that is free of errors, redundant, erroneous, or duplicate information within a dataset, and data cleaning can eliminate or correct such errors [21, 69–71]. In data cleaning, several steps are involved, such as sampling, processing abnormal values, discretizing data, and normalizing data [59]. With data sampling, highly accurate prediction models can be derived with fewer data, and abnormal values must be eliminated to ensure model accuracy. It is known that data discretization reduces the possible values for a continuous feature, while data normalization defines the data magnitudes at the same level to optimize several machine learning algorithms. It is possible that the dataset may be messy even after reducing noise, data redundancy, as well as abnormal values through these steps [72–74].

To ensure the representativeness and unbiased nature of the data used for model training, a multi-faceted approach is adopted. This includes defining clear objectives, selecting diverse and reliable data sources, using random sampling techniques, building a large and diverse dataset, preprocessing and cleaning the data, handling missing data appropriately, and detecting and mitigating biases. It must be acknowledged that achieving the complete elimination of biases remains a formidable challenge, however, a systematic and robust approach, coupled with regular assessments and monitoring, can substantially enhance the prospects of fairness and accuracy in the trained models.

### Feature Engineering

After collecting enough available data, appropriate features are obtained from the raw data and tasks, which is a key step in predicting targeted properties without redundancy [33, 75]. Features are also known as descriptors, which enable machine learning algorithms to perform at their best [76, 77]. Generally, an input descriptor can take the form of a number, a vector, a matrix, or a character string, and represents a series of input data assigned to the characteristic properties of the materials [78, 79]. If appropriate features are selected during feature extraction, the model can be made more understandable and accurate while reducing its dimensionality and complexity. It involves a cautious and comprehensive collection of reasonable descriptors [80–82]. In addition, feature selection not only simplifies, accelerates, and improves the interpretability of the learner models, but also allows for a deeper exploration of material characteristics through the use of feature engineering tools (Columbus, DeepDive, and Explorekit) [83–85]. Notably, the number of features should be considered before choosing features for a model [86–88]. Raw scientific data is converted into useful features that encode material information, including the density of state, Voronoi tessellations, chemical species, and one-hot encoded composition-based feature vectors [89–93]. For electrocatalytic materials, features related to the crystal and geometrical structure, element compositions, and electronic properties are used to predict and disclose destined properties containing activity, stability, and selectivity [19, 44, 94–99]. The electrocatalytic properties of materials can be predicted by converting features into descriptors, which should be broad and efficient in describing such properties [100]. However, the initial guess structure is a starting point, it may not invariably yield accurate structural descriptors. To enhance precision, a multifaceted approach often necessitates a synergy of additional strategies, iterative refinement, and optimization. For instance, in the landmark study by Chen et al. [101], a novel machine learning framework was devised to optimize local structures through a local machine- learning potential (MLP). This pioneering method enabled the extraction of precise structure descriptors, leading to the identification of 43 high-performance alloys as potential HER electrocatalysts from a pool of 2973 candidates. Several top candidates were further corroborated experimentally, with the AgPd alloy being systematically scrutinized using ab initio calculations under realistic electrocatalytic conditions to attest to the framework's accuracy. This approach epitomizes the fusion of computational efficiency and precision using optimized local MLP structural descriptors, offering a path forward in the design of high-performance electrocatalysts.

An electrocatalytic reaction, for example, is characterized by the bond energy formed between adsorbed hydrogen and the electrocatalyst, and this is expressible by the hydrogen adsorption energy (*E*_Had_), and the d-band structure. These two factors show the catalytic activity of the reaction [102]. Additionally, electronic and structural properties, such as Fermi level, work function, electron affinity, coordination numbers, and atomic radial distribution functions, are also important features [4, 20, 57, 96, 103–110]. With the rapid development of deep learning, automated feature engineering is now extensively utilized [111]. Using deep learning, computers are capable of learning features automatically from data and combining them during model construction, reducing the inadequacy of manual feature engineering [112]. A wide range of applications are possible with this technology including drug delivery, batteries, bioinformatics, and nanotechnology [113–116].

### Machine Learning Algorithms

**Fig. 2:**
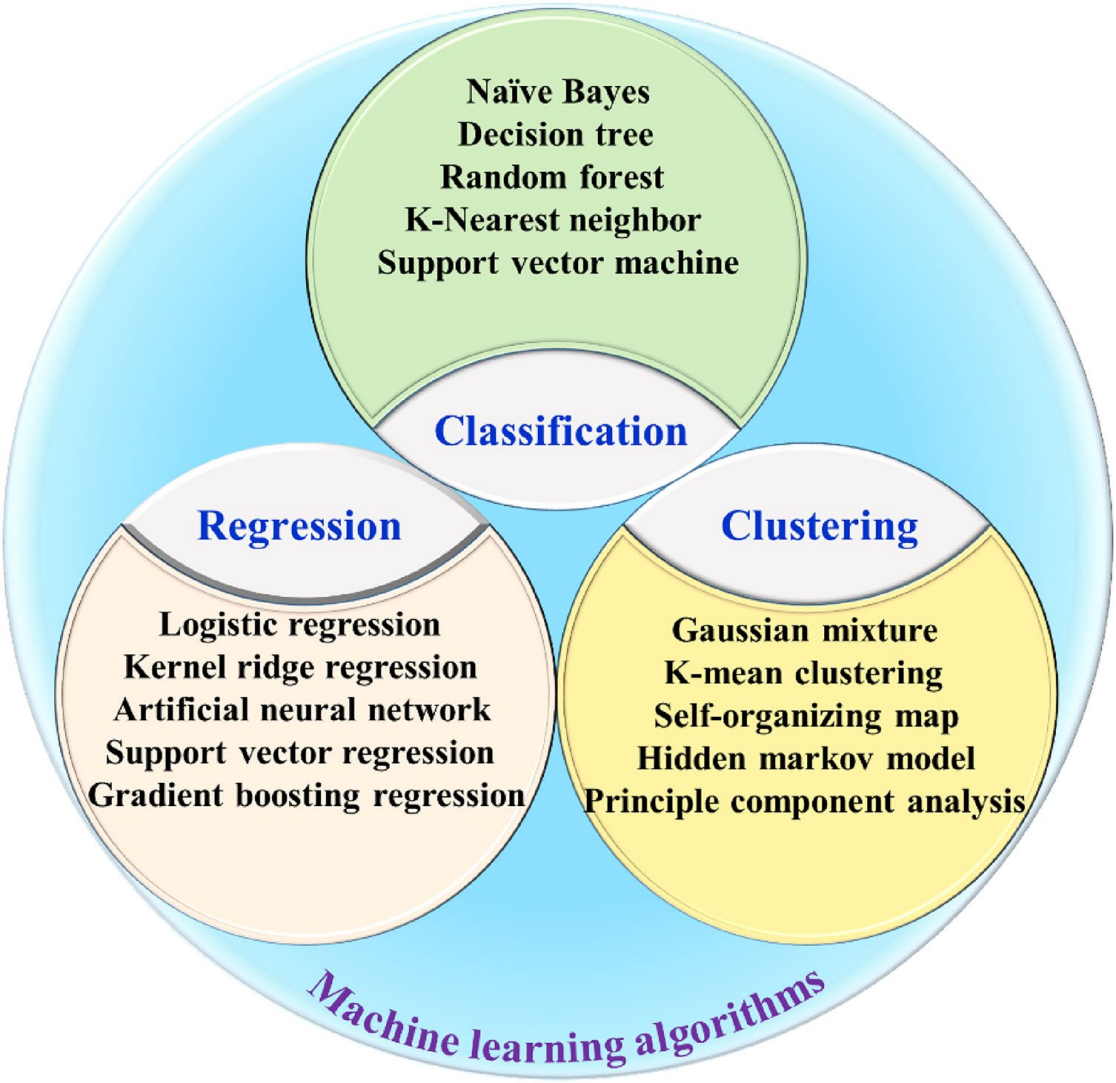
Typical machine learning algorithms in electrocatalyst design

Machine learning is an efficient and powerful strategy towards predicting electrocatalytic performance due to its ability to correlate input features (properties of the electrocatalyst) with output parameters (electrocatalytic performance) [117, 118]. The selection of an appropriate machine learning algorithm is crucial for achieving high prediction and generalization ability. Several mathematical theories have been applied to the creation of machine learning algorithms, including Markov chains, least squares methods, and Gaussian processes. In materials science, machine learning algorithms can be grouped into three types: classification, regression, and clustering (Fig. 2), with each algorithm offering unique advantages and limitations. While the details of each algorithm have been reviewed and summarized elsewhere [31, 41, 94], we present here some representative algorithms that have been employed for establishing deep structure–activity relationships.

Material science has become increasingly interested in kernel-based algorithms because they are capable of handling complicated regression issues beyond the capabilities of simple linear models. As a result of the kernel trick, ridge regression, and classification are often combined to yield kernel ridge regression (KRR), which can predict the electrochemical activity of electrode materials according to their structural characteristics [119]. As a binary classification and regression algorithm, the support vector machine (SVM) classifies training data into two distinct classes with a low error by using a hyperplane. For instance, SVM can classify high-entropy alloy solid solution phases across feature ensemble sizes [20]. In both SVM and KRR, the hyperparameter settings have a significant impact on performance. SVM takes less time in prediction, while KRR spends less time to fit medium datasets. Despite this, the training time of SVM and KRR cannot be exclusively attributed to the dataset size. Rather, it emerges as a function of various intertwined factors, encompassing the complexity of the algorithms, the specific attributes of the dataset, and potentially other underlying considerations that contribute to the overall computational demand. An artificial neural network (ANN), which simulates biological neural networks, is often included in machine learning models. Because of its self-learning and self-adaptive abilities, it can adjust its internal structure as it learns and adapts to external information. As a reliable algorithm, it has been utilized to forecast the rest usable lifetime of Li-ion batteries online and screen catalysts for their effectiveness as a reliable algorithm for large datasets [58, 120, 121]. Moreover, Rice and coworkers utilized a high-dimensional neural network, along with sampling methodologies and DFT calculations, to explore the efficiency of the hydrogen coupling reaction at the H_2_O/Pt (111) interface [122]. The aim was to obtain a detailed atomistic understanding of how the presence of an aqueous medium impacts the structure and reactivity of the HER. By using cutting-edge tools and techniques, the research team was able to gain valuable insights into the underlying mechanisms of the reaction, which can inform the development of more effective HER electrocatalysts. This approach provides a detailed and comprehensive picture of the hydrogen coupling reaction at the interface, which can serve as a basis for further research and advancement in the field of the HER. ANN is capable of addressing complex nonlinear problems with large datasets, but requires a deeper understanding of machine learning, which is more difficult to operate than SVM and KRR owing to the weight setting [123, 124]. Furthermore, the K-nearest neighbor algorithm is applied extensively to identify and predict materials with excellent performance [125–127]. In the case of large datasets, however, it may require a considerable amount of time and memory. Also, other algorithms, like logistic regression and decision trees, are utilized, which require strict mathematical reasoning, strong interpretation, and fast running speeds, making them suitable for small datasets [128, 129].

### Model Optimization

Data sets are crucial to evaluating the stability and performance of machine learning models in material research. Accurately dividing these data sets into training and test sets is a critical step in assessing the model's effectiveness. Typically, 80% of the original data is found in the training set, while a test set includes the remainder. The process of evaluating errors in both the training and test sets provides valuable insight into the extent of underfitting or overfitting of a model. Overfitting occurs when training errors are much smaller than validation errors, whereas underfitting occurs when both errors are high but the gap between them is small. To prevent overfitting or underfitting within the models, several strategies can be employed. These encompass techniques such as cross-validation to assess how the results of a statistical analysis will generalize to an independent dataset, regularization to add some form of penalty to the loss function, feature selection to choose relevant predictive variables, increasing training data to promote a richer understanding of underlying patterns, early stopping to terminate training when validation performance deteriorates, ensemble methods to combine predictions from multiple models, and data augmentation to increase the diversity of the training set. It is important to note that the selection of an appropriate strategy depends on the specific problem, as well as the characteristics and nuances of the underlying data. Through careful and considered application of these techniques, it is feasible to mitigate the risks of overfitting and underfitting, thereby enhancing both the performance and the generalizability of the model. For preventing overfitting and effectively using limited data, cross-validation methods are commonly utilized. Typically, these methods involve running the model multiple times with different data sets, and splitting each set in a way that maximizes the use of the data available in each set. There are several methods of cross-validation, including *k*-fold cross-validation, repeated random subsampling analysis, Bootstrap cross-validation, and leave-out cross-validation [130–133]. Machine learning models can be assessed and compared by utilizing various metrics, such as mean-absolute-error (MAE), mean squared error (MSE), and root mean square error (RMSE) [134].

## Machine Learning Application for the HER

**Fig. 3:**
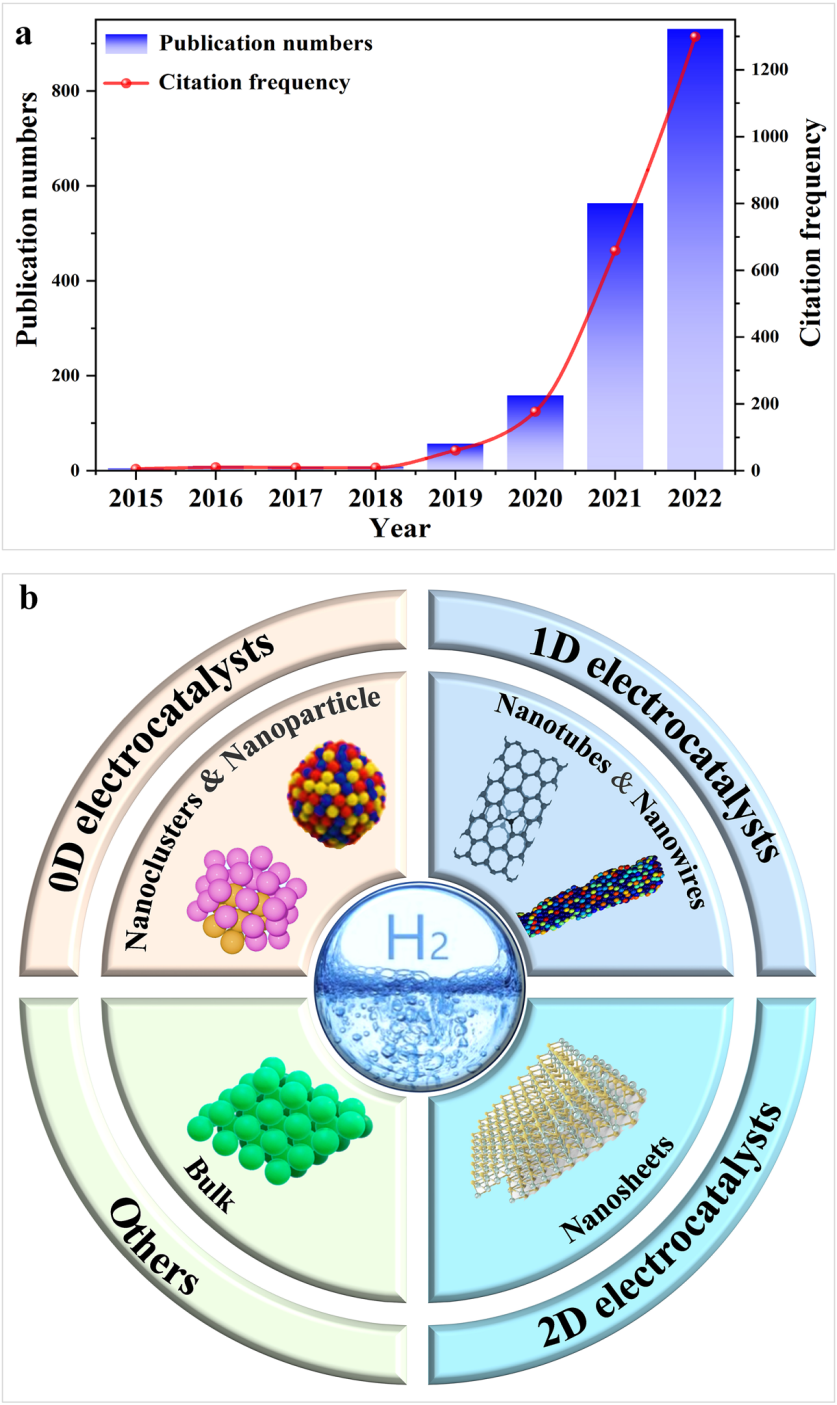
**a** Histograms of the number and citation frequency of relevant articles were retrieved with the keywords "machine learning" and "hydrogen evolution reaction" from the Web of Science database. **b** Machine learning for designing various electrocatalysts including 0D electrocatalysts, 1D electrocatalysts, 2D electrocatalysts, and others

Materials science and engineering researchers are committed to developing materials that have desired properties rationally and efficiently [31, 135–137]. However, traditional trial-and-error methods have significant drawbacks, including high costs and time investments, which can impede progress and limit the exploration of advanced materials [138–140]. As machine learning advances, researchers can develop models that can make accurate predictions with greater efficiency than pure DFT calculations [141]. This revolutionary trend has greatly enhanced the development of the HER, as evidenced by the publication of over 1741 related articles related to machine learning methods to the HER during the past 8 years (Fig. 3a). In this section, an in-depth discussion of how machine learning techniques can be applied to the advancement of low-dimensional electrocatalysts will be presented (Fig. 3b), including noble metals, metal alloys, MXenes, carbon-based materials, metal phosphides, metal dichalcogenides, and others, and examine their performance. In the research for the HER, machine learning techniques play an essential role, and their application will significantly accelerate the progress toward developing a rational design of advanced electrocatalysts, thereby speeding up the research process.

### 0D Electrocatalysts

**Fig. 4:**
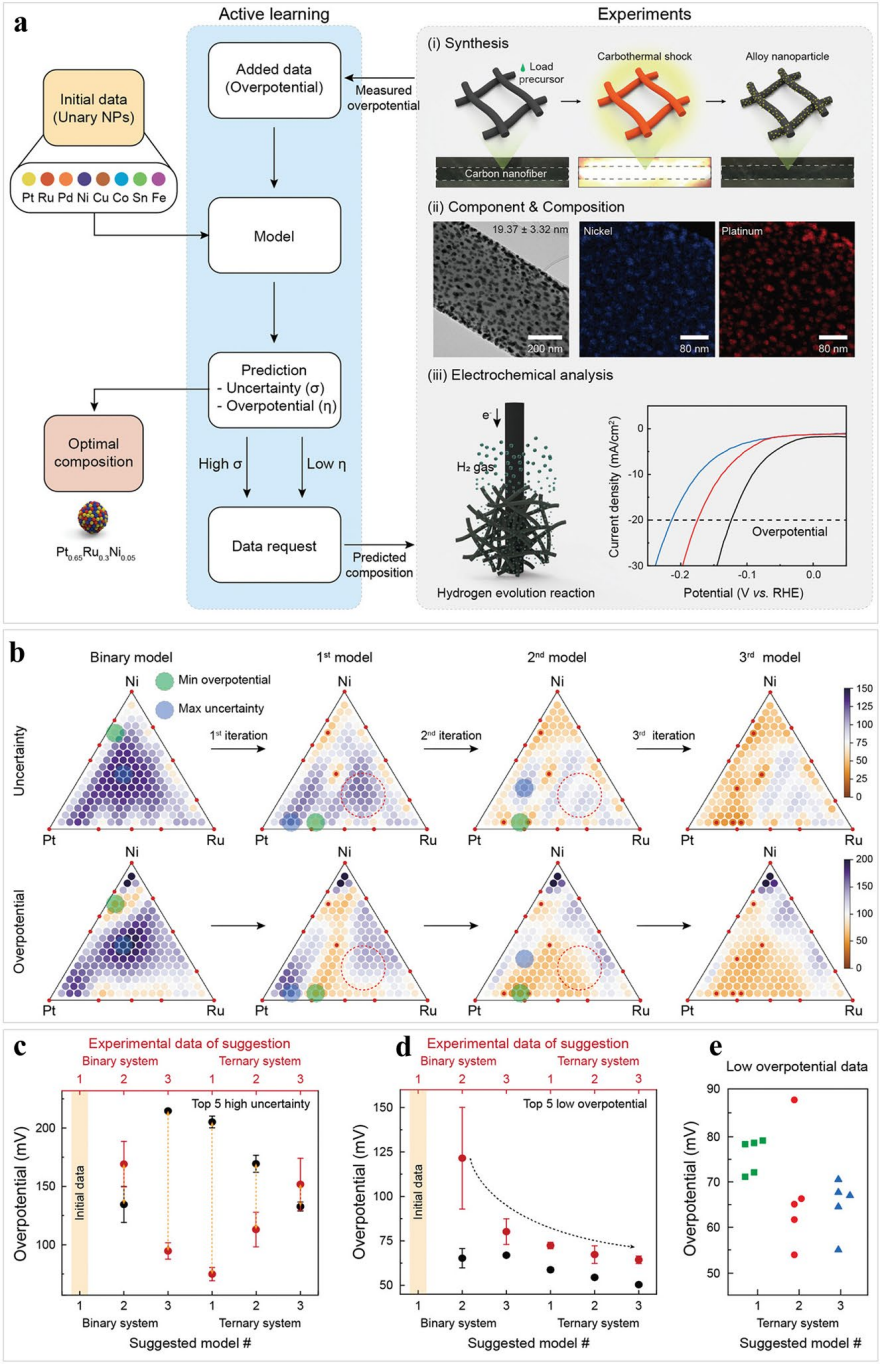
Exploring MMA electrocatalysts via active learning and experiments. **a** Process of developing MMA electrocatalysts with small overpotential. **b** Overpotential and uncertainty of the Pt-Ru-Ni catalysts. At certain points during the iteration process, the changes in predictions, which were not influenced by adding any additional data, were symbolized by the red dotted circles. **c** Graph showing the overpotential of the top-five high-uncertainty points (THP). The corresponding results were marked by black and red circles, respectively. Any differences between the two results were highlighted by orange arrows to help provide a clear comparison. **d** Plot of the THP changes in overpotential. **e** Scatter-plotted ternary data points. Reproduced from Ref. [142] with permission from John Wiley and Sons

It is one of the crucial issues to discover excellent multi-metallic alloy (MMA) electrocatalysts that contain an optimal component and composition for the HER [80]. Recently, through the use of active machine learning in conjunction with experimentation, an optimal MMA catalyst was found. The model resulting from the experiment was successfully built solely by using the composition of the mixed precursor as input data, which resulted in an enhanced electrocatalytic performance (Fig. 4a) [142]. Interestingly, the strategy could be applied extensively by adjusting an electrocatalyst composed of an optimal component. As displayed in Fig. 4b, only binary data were used to train the model, which resulted in high uncertainties. A significant reduction in the uncertainty of the Pt-Ru-Ni sample has been achieved by updating the model in an extensive range of compositional possibilities with a minimum overpotential. Also, from Fig. 4c, d, we can see that there is the less expected improvement in model performance with additional iterations than at the beginning of the loop. Furthermore, as presented in Fig. 4e, several points were located. Consequently, the HER overpotential of an optimal Pt_0.65_Ru_0.30_Ni_0.05_ electrocatalyst was 54.2 mV, even exceeding a pure Pt catalyst. This work simplifies the challenges associated with identifying efficient catalyst components and compositions and can be extended to other catalytic reactions.

**Fig. 5:**
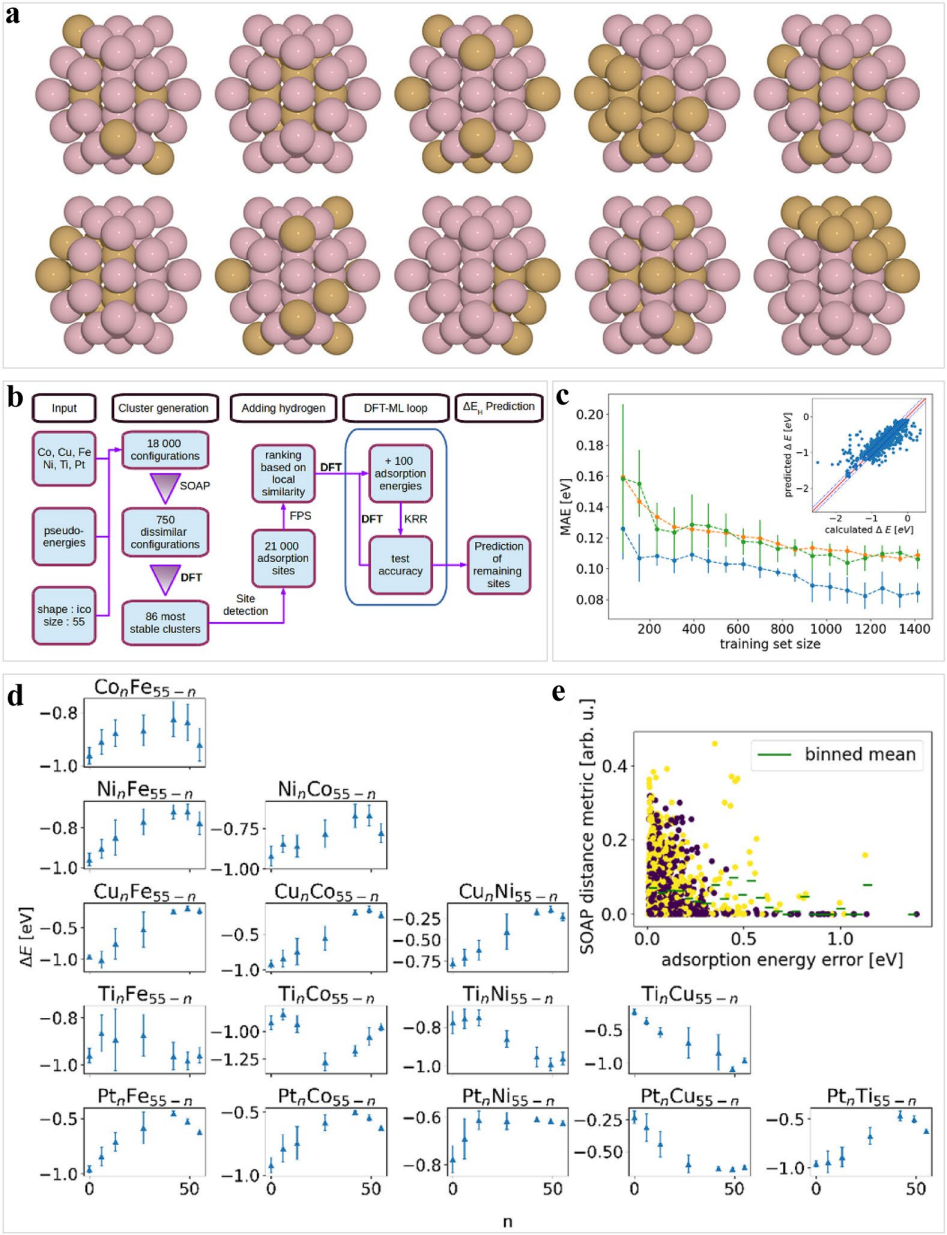
Machine-learning-assisted investigation of E_Hads_ on bimetallic nanoclusters. **a** Cu_13_Co_42_ clusters include core–shell, segregated, ordered, and random structures. **b** A workflow outline exhibits the process from the formation of a cluster to the prediction of the *E*_Had_ distributions. **c** Learning curve of KRR, the inserted image shows the calculated versus predicted *E*_Had_ of 1767 DFT calculations. **d** Predicted E_Had_ distribution. **e** Evaluation of machine learning accuracy in the presence of adsorption site drift and surface reconstruction. Reproduced from Ref. [147] with permission from American Chemical Society

In addition, nanoclusters possess alterable catalytic activity at the nanoscale, rendering them promising candidates for catalysis applications [143–146]. Nevertheless, computational screening methods face difficulties owing to numerous relevant atomic sites in nanoclusters [102]. To address this issue, new techniques for efficient exploration are required. In this regard, as a means of replacing noble metals with eco-friendly and cost-effective HER electrocatalysts, Cu-based alloy nanoclusters with 7924 candidate structures were screened (Fig. 5a, b) [147]. High-throughput DFT computations and machine learning were employed to identify the most stable core–shell configurations for Cu_*55-n*_M_*n*_ (M = Co, Ni, Ru, and Rh, and *n* ≤ 22) nanoclusters. A descriptor that eliminated the burdensome computations was developed to assess the activity of the HER on nanoclusters. Subsequently, an ANN was trained with a large DFT database to quickly and precisely predict the *E*_Had_ on the surface of the nanoclusters (Fig. 5c–e). This work provided an effective strategy to quickly discover HER electrocatalysts with great potential based on metal alloy nanoclusters. Additionally, machine learning was found to lower the consumption of modeling various adsorption site structures aided by descriptors [148]. Advanced structural descriptors, including Many-Body Tensor Representation, Smooth Overlap of Atomic Positions, and Atom-Centered Symmetry Functions, were investigated by Jager et al. for predicting the Gibbs free energy of hydrogen adsorption (Δ*G*_H*_) on the nanoclusters [102]. The accuracy of the descriptors in KRR was evaluated by potential energy scans of hydrogen on the nanocluster surfaces. Analyzing the data sets of 91 molybdenum disulphide nanoclusters and 24 copper–gold nanoclusters, machine learning could reduce MAE by learning diverse nanoclusters simultaneously as opposed to sequentially. Furthermore, they observed a marked reduction in the fitting of potential energy surfaces when data from different nanoclusters were merged. Also, their group demonstrated a machine learning-based workflow for nanocluster configurations and adsorption energy screening [149]. The results presented that the adsorption was exemplified on the HER, and the maximum of the d-band Hilbert-transform *ϵ*_u_ was associated with the *E*_Had_ at the nanocluster level.

### 1D Electrocatalysts

**Fig. 6:**
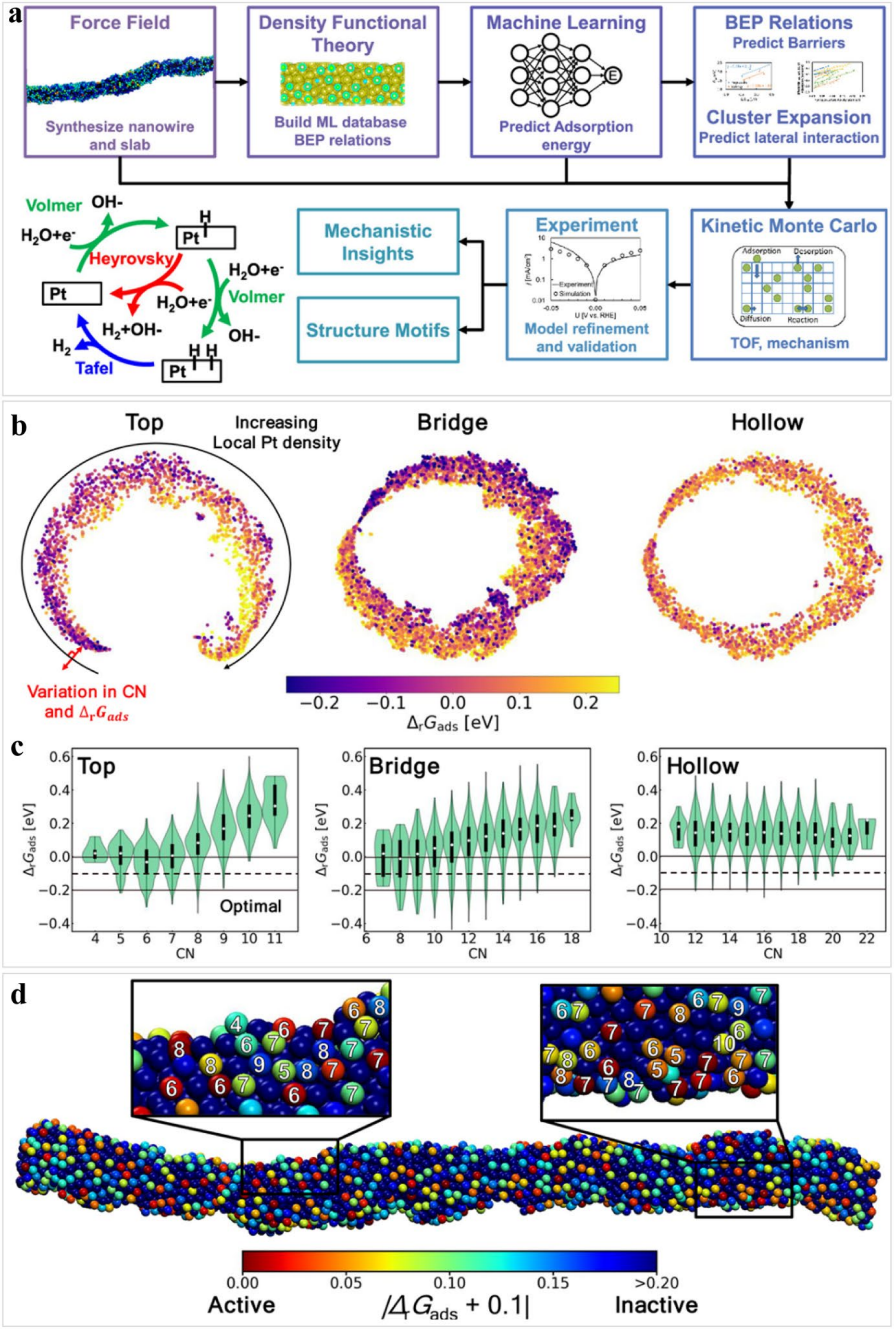
Investigating jagged Pt nanowires via end-to-end simulation. **a** Flowchart for the jagged Pt nanowire using end-to-end simulation. **b** Identifying active sites with ΔrG_ads_ values towards the top, bridge, and hollow sites. **c** Plots of ΔrG_ads_ values versus coordination numbers. **d** Visualization of Pt nanowires with an optimal ΔrG_ads_. The magnification indicates that the low coordination numbers of Pt atoms possess suitable ΔrG_ads_ values. Reproduced from Ref. [154] with permission from American Chemical Society

Through ab initio simulations of reaction and activation energies, the HER kinetics of various electrocatalysts have been extensively studied [150]. The effect of the water static layers, hydrogen-bond networks, adsorbed species, and electric double layers increase the uncertainties in the energetics related to the DFT methods [151–153]. Although the Tafel slope and the *E*_Had_ are frequently applied to study the HER kinetics, methods for revealing the HER kinetics remain challenging. Therefore, simulating and interpreting kinetics with adequate rate expressions is an essential step for understanding HER mechanisms [122]. Gu et al. utilized an end-to-end avenue to aid in simulating the kinetics of jagged Pt nanowires by machine learning multiscale method (Fig. 6a), which was consistent with the experimental results in alkaline media [154]. They demonstrated that the optimal Δ*G*_H*_ value in alkaline solution for the overall rate was lower than that of acidic condition, and the jagged Pt nanowires exhibited an auto-bifunctional mechanism, namely, protons were adsorbed on the stronger binding sites and hydrogen was activated on weaker binding sites. Meantime, unsupervised machine learning model results indicated that Δ*G*_H*_ was interrelated with the coordination number, as well as the sites with CN ≤ 7 showed great activity (Fig. 6b, c). Additionally, as displayed in Fig. 6d, the plot of the Δ*rG*_ads_ values and coordination numbers of top sites from the Pt nanowire proportion indicated that exposed sites with low coordination were nearer to the optimal Δ*rG*_ads_ value. This work is conducive to comprehending complex kinetic processes on the nanoscale and HER mechanism.

**Fig. 7:**
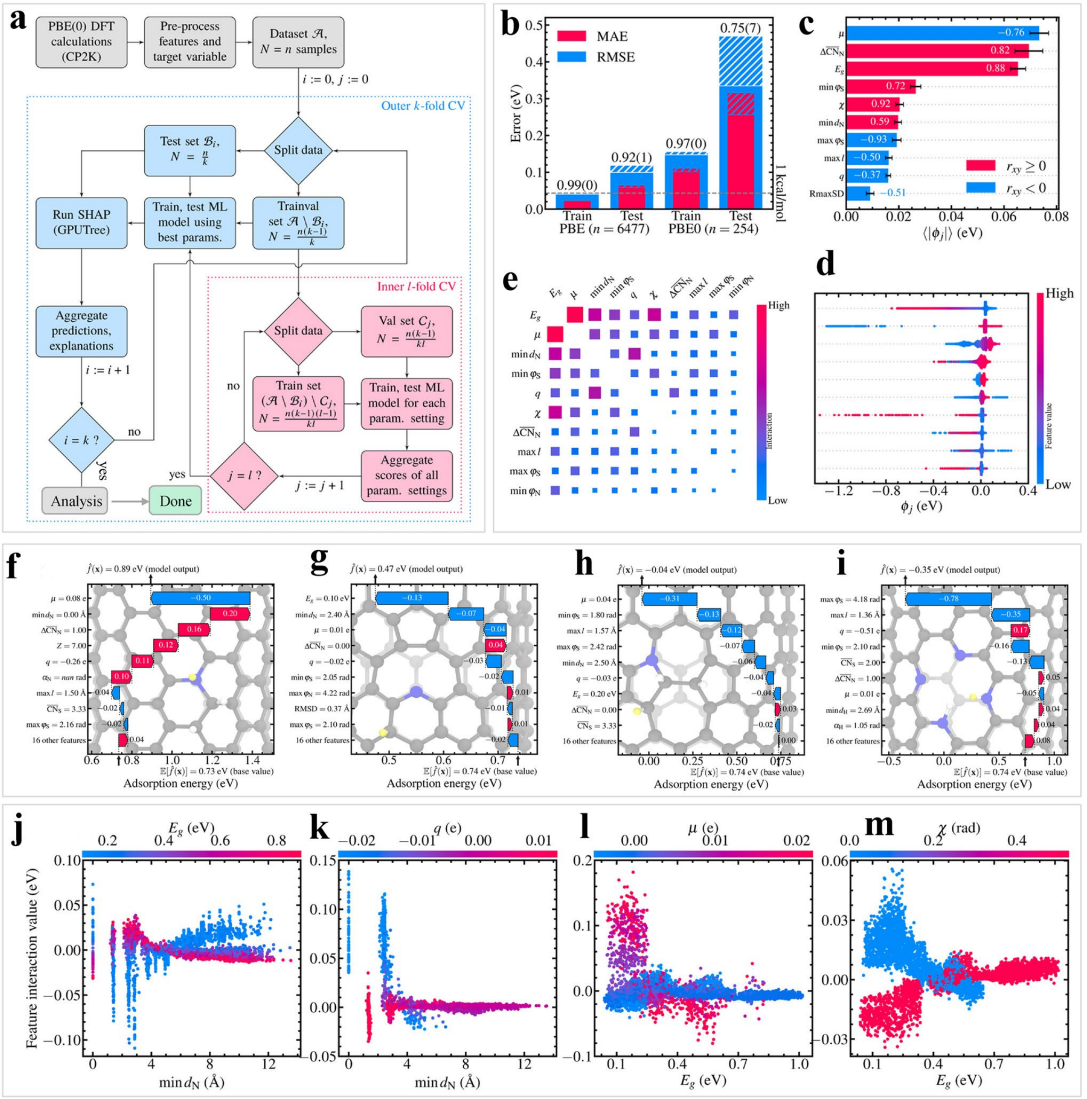
Hydrogen adsorption on defective NCNTs was interpreted through machine learning. **a** Workflow utilized machine learning and SHAP analysis for defective NCNTs. **b** Unbiased generalization performance of the RF models. **c** The importance of ten features in the predictions of adsorption energy. **d** SHAP values for the important features. **e** Measuring SHAP strong interaction effects of the ten features. Partial descriptions for adsorption at **f** (8,8) graphitic, **g** (14,0) N_1_V_1_-pyridinic, **h** (14,0) N_1b_SW-pyrrolic, and **i** (8,8) N_4_V_2_-pyridinic dopant configurations are illustrated. Pairwise SHAP interaction effects between **j** the dopant-adsorption site separation and the NCNT energy gap, **k** the dopant-adsorption site separation and the residual charge on the adsorption site, **l** the energy gap and the spin polarization on the adsorption site, as well as **m** the energy gap and the NCNT chirality. Reproduced from Ref. [156] with permission from American Chemical Society

Adsorbed hydrogen, as an intermediate, plays a crucial role in the HER [155], and *E*_Hads_ are commonly served as descriptors in machine learning. For example, Kronberg et al. investigated and interpreted the hydrogen adsorption performance on defective nitrogen-doped carbon nanotubes (NCNTs) using DFT simulations and Shapley additive explanations (SHAP) analysis based on machine learning (Fig. 7a) [156]. The authors achieved significant results, as evidenced by the MAE and RMSE results presented in Fig. 7b, which showed highly accurate predictions on the training set, attaining a chemical accuracy with an *R*^2^-score of 0.99. According to Fig. 7c–e, the attributions of each feature were summarized based on the mean magnitude of the SHAP values. More importantly, by using the SHAP strategy, they examined several chemical, structural, and electronic properties of around 6500 different NCNTs in relation to hydrogen adsorption. Some factors are considered to be responsible for the increased hydrogen adsorption strength of the high spin polarization as well as the dopant-adsorption sites, narrow gap between the highest occupied and lowest unoccupied molecular orbitals, and diverse angles and coordination effects. The method also allowed for the investigation of catalyst durability problems, as indicated by the breakage of pyrrolic nitrogen bonds, highlighting the balance between defect- and dopant-caused activation and extreme instability (Fig. 7f–i). Moreover, four feature pairs interactions were investigated (Fig. 7j–m). The results showed that different features would affect each other, potentially impacting the model output. Prominently, it has been shown that analogous features could affect the hydrogen adsorption and HER performance on defective NCNTs, which may exert a positive influence on the future design of HER electrocatalysts.

### 2D Electrocatalysts

**Fig. 8:**
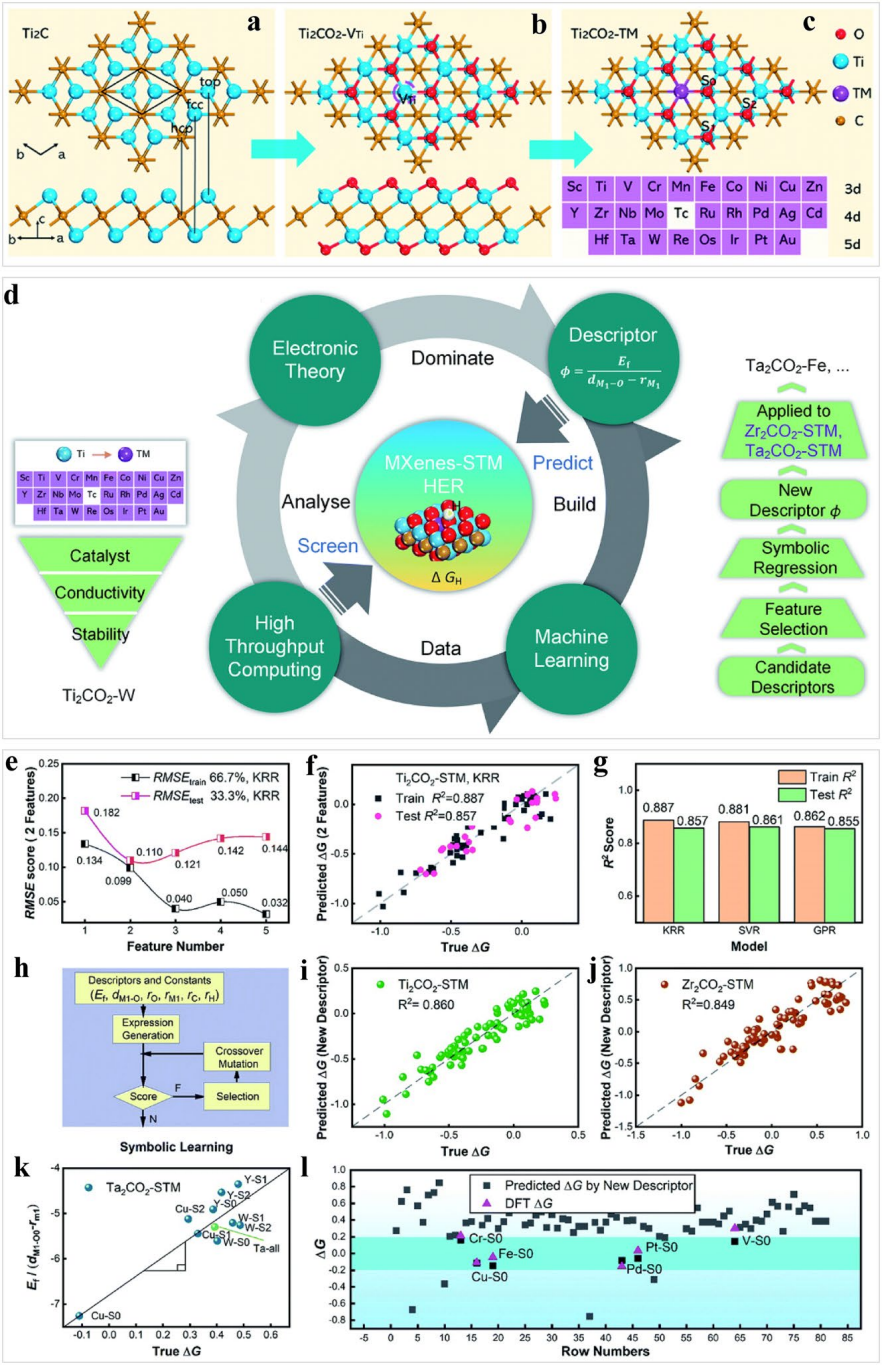
A descriptor for designing 2D MXene HER electrocatalysts. **a** Ti_2_C structure containing top, fcc, and hcp, representing the O adsorbed sites. **b** Defective Ti_2_CO_2_ with Ti vacancy. **c** Doped model Ti_2_CO_2_-STM and TM = 3d, 4d, and 5d metals with single atom. S_0_, S_1_, and S_2_ correspond to O positions for H adsorption. **d** Overall flow of the high throughput computation and machine learning. **e** Descriptor performance in the KRR. **f** R^2^ of two important descriptors for KRR, and **g** other models. **h** Genetic programming processing. **i**, **j** The prediction performance of Ti_2_CO_2_-STM and Zr_2_CO_2_-STM with the new descriptor. **k** Fitting coefficient definition for Ta_2_CO_2_-STM. **l** Validation and new catalyst screening in Ta_2_CO_2_-STM. Reproduced from Ref. [165] with permission from Royal Society of Chemistry

The use of machine learning to screen large material databases for appropriate electrocatalyst properties offers significant advantages in terms of reduced experimental cycles and costs, as well as addressing the challenge of material selection [157]. 2D materials with more active sites show great potential in large-scale hydrogen production, which is expected to replace Pt catalysts [158–162]. Nevertheless, the rapid discovery of high-performance 2D HER catalyst is greatly depressed owing to the long experiment cycles and high costs associated with high-throughput calculations for adsorption energies [163]. Particularly, MXene-based materials are gaining considerable attention because of their potential use in the HER [5, 155, 164]. For instance, the 2D metal carbide semiconductor Ti_2_CO_2_ MXenes with a cost-effective basal plane has been used as an electrocatalyst for the HER, and the corresponding HER performance could be modulated by immobilizing single transition metal (STM) atoms onto Ti vacancies (Fig. 8a–c) [165]. Accordingly, machine learning methods were used to identify 27 different MXenes Ti_2_CO_2_-STMs and 81 HER catalytic active sites using a facile descriptor, which was then applied to explain the trends in Ti_2_CO_2_-STM HER catalysis and search for other highly active HER catalysts (Fig. 8d). As exhibited in Fig. 8e, the training error decreased unremittingly before overfitting occurred. Subsequently, two descriptors of *d*_M1–O_ and *E*_f_ were selected for constructing a machine-learning model with KRR towards the HER (Fig. 8f, g). From the symbolic regression, which used *d*_M1–O_, *E*_f_, covalent radii *r*_O_, *r*_Ti_, *r*_C_, and *r*_H_ as input, and the expression for Δ*G*_H*_ could be obtained (Fig. 8h, i). To verify the validity of the descriptor, the Δ*G*_H*_ values of Ti_2_CO_2_-STM and Zr_2_CO_2_-STM were calculated and showed small *R*^2^ scores. (Fig. 8j, k). A fitting coefficient was defined to further prove the descriptor's ability to explore promising HER catalysts, which successfully predicted all 81 points of Ta_2_CO_2_-STM, as exhibited in Fig. 8l. The corresponding results implied that STM doping not only optimized the Δ*G*_H*_ but also transformed semiconductors into conductors, and hybridization of the p-d orbitals between STM and C/O resulted in the rearranging of electrons close to the Fermi level, improving HER performance. In addition, the adsorption energy of hydrogen on the active sites is a primary factor determining HER activity [19]. For instance, as compared to traditional methods, Zheng et al. showed that machine learning models were more effective in predicting the Δ*G*_H*_ of S-terminated and bare MXenes [166]. Four machine learning models, including Elman ANN, support vector regression, KRR, as well as random forest algorithms (RFA), were selected to predict Δ*G*_H*_. According to the results, Δ*G*_H*_ values predicted via the RFA had a high-level accuracy, and testing RMSE was 0.27 eV. Furthermore, the Δ*G*_H*_ values of Os_2_B- and S-terminated Sc_*n*+1_N_*n*_ (*n* = 1, 2, 3) approached zero within a wide hydrogen coverage, resulting in excellent HER. In addition, HER performance was improved through the regulation of antibonding states by S functional groups. Moreover, the successful development of machine learning models coupled with DFT calculations has enabled the prediction and design of HER electrocatalysts from numerous bare and single-atom doped B-based MXenes [167]. Utilizing a support vector algorithm with structural and elemental features, the Δ*G*_H*_ values were computed for 271 B-based MXenes. This approach allowed for the prediction of diverse active catalysts, including Co/Ni_2_B_2_, Pt/Ni_2_B_2_, Co_2_B_2_, Os/Co_2_B_2_, and Mn/Co_2_B_2_ with small Δ*G*_H*_ values. Among these materials, Co_2_B_2_ and Mn/Co_2_B_2_ were identified as the most stable electrocatalysts, as they exhibited small ΔG_H*_ over a broad hydrogen coverage. This study indicated that machine learning models were exciting tools for developing advanced HER electrocatalysts. Besides, the integration of high-throughput DFT and machine learning models have garnered significant attention for determining activity trends in 2D MXenes and guiding HER catalyst design [168]. This approach has led to the screening of 188 ideal HER catalysts with high mechanical and thermal stability from 2520 candidates that can be experimentally synthesized. Notably, 110 of these 2D MXenes exhibited exceptional thermos-stability and HER activity, surpassing even the ideal Pt catalyst. Additionally, extensive accuracy was achieved in predicting the HER activity for 2D MXenes using the AdaBoost ensemble learning method, suggesting that this approach is a promising strategy for developing superior 2D MXene catalysts.

In recent studies, it has been demonstrated that certain 2D graphene and graphdyine-based SACs present exceptional activity for the HER [80, 169–173]. To explore the efficient HER catalysts, Fung et al. applied machine learning to systematically screen SACs anchored on 2D graphene [174]. It has been demonstrated that the dimensionality and size of the substrate can affect hydrogen adsorption on metals, resulting in modifications in the electronic structure of the metal. Furthermore, when compared to graphene-based SACs including V, Rh, and Ir, nanographene containing these elements displayed improved HER performance. This strategy not only facilitates the rapid exploration of better HER catalysts by modulating and doping the nanographene support but can also be easily extended to other catalytic reactions. Analogously, 104 graphene-supported SACs and their applications for the HER were described using machine learning models, along with a quantitative evaluation of their importance [175]. The outstanding catalytic activities of these machine learning-recommended SACs were documented by DFT calculations, and the best-performing catalysts delivered an ultralow overpotential of 3 mV for the HER, exceeding the noble metal Pt. As part of this machine learning approach, factors such as geometry optimization, total energy calculation, and analysis of reaction pathways were not included. Significantly, this method can be employed to screen and construct other catalysts for N_2_ and CO_2_ reduction reactions as well. Moreover, Sun et al. employed machine learning models to perform theoretical calculations and design potential graphdyine (GDY)-atomic catalysts (AC) electrocatalysts, while also investigating the HER mechanism, reaction pathway, and adsorption energies by considering multiple parameters [176]. This study represents the first comprehensive calculation of the *E*_Hads_ on different active sites of GDY-M (transition/lanthanide metals) during the HER. The electronic structures of the GDY-AC electrocatalysts were also evaluated and screened out. Additionally, the authors utilized the bagged-tree approach as the machine learning algorithm, based on the fuzzy model for data separation, to predict the *E*_Hads_ for various AC systems. This work provides innovative theoretical comprehension and direction for GDY-based AC for the HER.

**Fig. 9:**
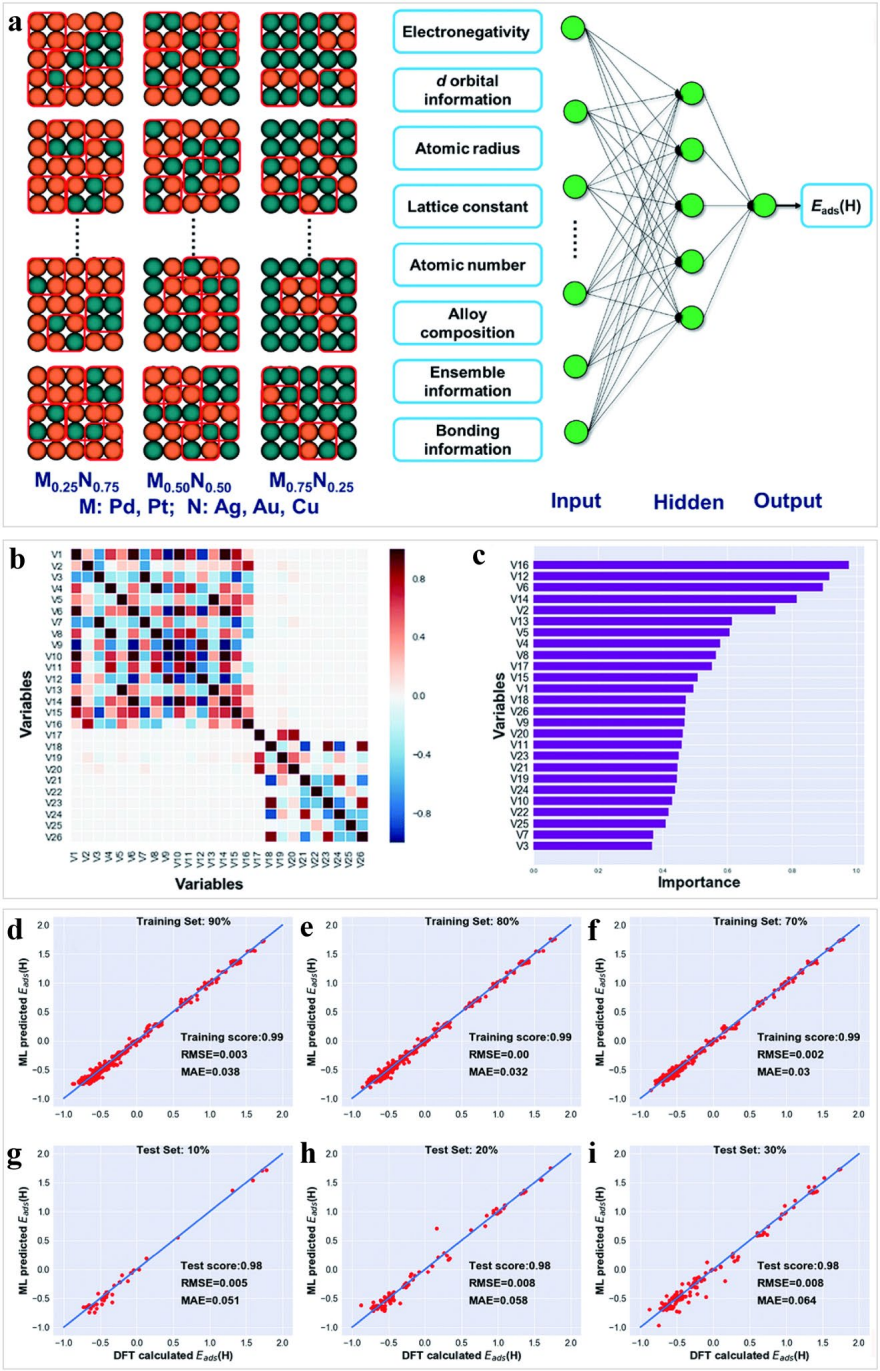
Application of machine learning in alloy electrocatalysts. **a** Illustration of (100) bimetallic alloys with the random sampling method. The red squares indicate the unique H adsorption environment created by a fourfold ensemble. **b**, **c** Feature analysis. Typical results of DFT calculated versus predicted *E*_Had_, **d**, **e** The ratio is nine to one between training and testing, **f**, **g** eight to two, and **h**, **i** seven to three. Reproduced from Ref. [178] with permission from Royal Society of Chemistry

In addition, recent reports have highlighted other metallic alloy catalysts with 2D active surfaces. Bimetallic alloy catalysts containing dominant (100) facet exhibited decent catalytic performance and durability [177–180]. However, there has been less attention paid to the alloying effects at (100) surfaces compared to surfaces that are closely packed. By means of high-throughput DFT calculations and machine learning methods, Li et al. demonstrated that certain (100) surfaces alloyed with strong-binding metals (Pd and Pt) and weak-binding metals (Ag, Au, and Cu) are capable of operating as HER catalysts in acidic solutions (Fig. 9a) [178]. To systematically predict the HER activity of other bimetallic alloys, a machine-learning model was developed by the DFT-calculated database. Before machine learning modeling, feature selection was conducted (Fig. 9b, c), determining the most important input variables to predict H binding energy. Subsequently, a BPNN model was constructed by the selected features. The results, shown in Fig. 9d–i, indicated excellent accuracy for both the training and test sets. For different combinations, the scores of the training and test all surpassed 0.97 with small RMSEs (less than 0.01). This study found that Pd_*x*_Ag_1−*x*_ and Pd_*x*_Au_1−*x*_, due to their highly active fourfold ensembles, showed encouraging HER activities on their surfaces (100), relative to their monometallic counterparts. It is anticipated that this work will offer valuable direction in designing and fabricating bimetallic alloys for the HER.

### Others

In the electrocatalysis field, the active sites on the surfaces of 0D, 1D, and 2D materials have been found to exhibit effective catalytic performance for the HER [181–183]. However, the bulk nature of these materials leads to relatively low numbers of active sites, which limits their potential as compelling catalysts, thus constraining the exploration of HER performance in bulk materials using machine learning methods. Nevertheless, some bulk materials, including metal–organic framework (MOF) and porous graphene, possess a multiscale porous structure that can also act as catalytic active sites [140]. For instance, Li et al. developed a ruthenium-adapted MOF electrocatalyst that distributed atoms at an atomic level. This electrocatalyst can efficiently adjust the MOF metal center's electronic structure, and it convincingly catalyzes the HER reaction [184–186]. However, the theoretical calculations involve multi-dimensional interactions of larger systems, resulting in very few studies by means of machine learning. This presents an opportunity to explore and better understand the electrocatalytic performance of these materials coupled with machine learning in the future.

## Conclusions and Perspectives

**Fig. 10:**
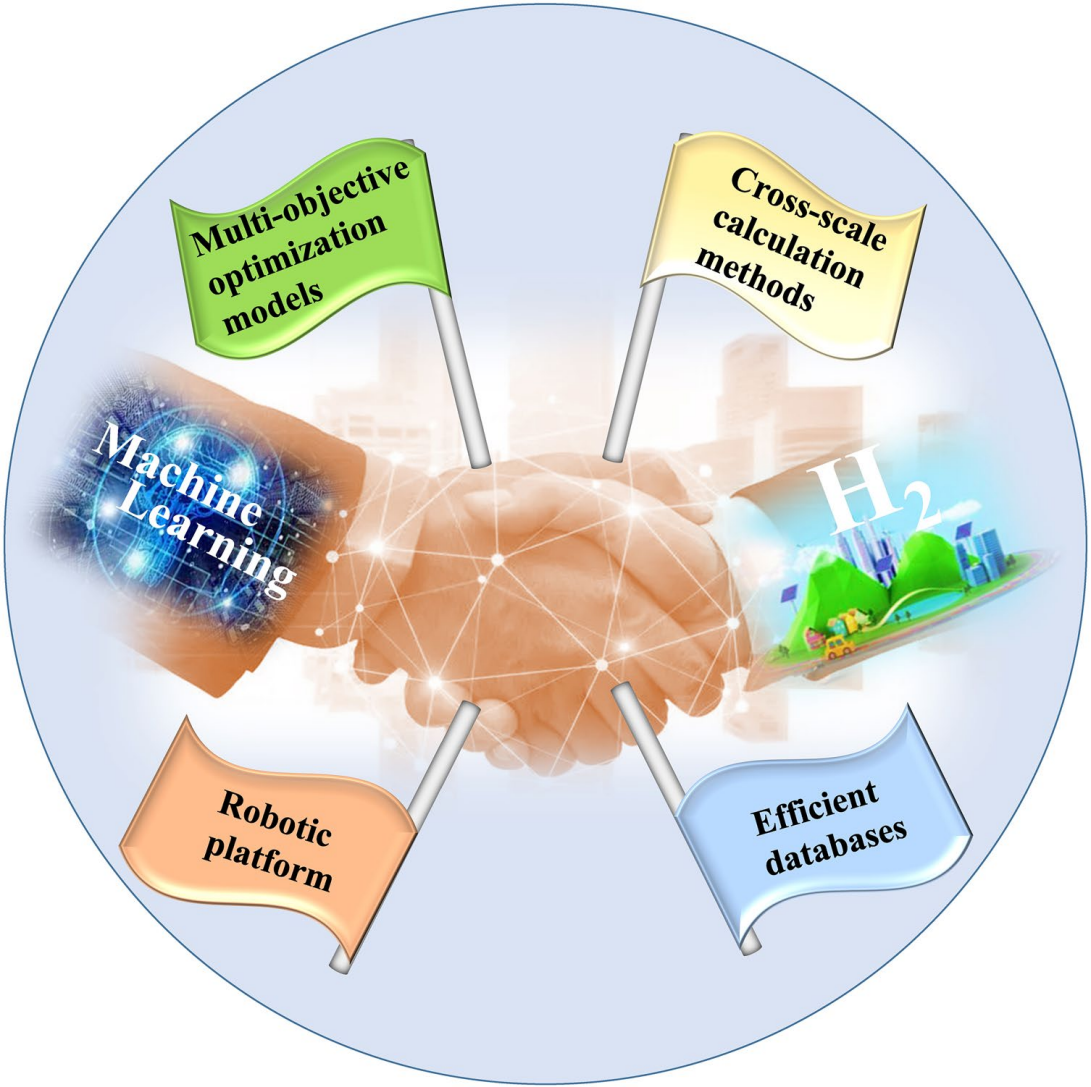
Perspectives of machine learning for the HER

Without large-scale trial-and-error experiments or theoretical calculations, machine learning can efficiently explore electrocatalysts and predict their properties. This technique has been widely used to rapidly and effectively screen low-dimensional electrocatalysis systems, providing a fundamental understanding of electrochemical reactions from the atomic scale. By elucidating the development of input characteristics, structures, and descriptors, as well as learning algorithms for performance prediction and electrocatalyst screening, this work highlights the advances in machine learning methods for investigating, comprehending, and optimizing the HER. Despite the great progress that has been achieved in the HER through the implementation of machine learning techniques, there are some trends in developing high-performance HER electrocatalysts via machine learning, as illustrated in Fig. 10.

1. In the field of electrocatalysis for the HER, the development of efficient and effective datasets is crucial. Accurate prediction of the activity, selectivity, and stability of different electrocatalysts is essential in designing superior materials for HER electrocatalysis. Machine learning can aid in this process by analyzing large datasets and identifying patterns and correlations between various properties of the electrocatalysts. An effective dataset for HER electrocatalysts should include a diverse set of electrocatalysts with varying properties, such as composition, morphology, crystal structure, and surface area, among others. The dataset should also include precise experimental measurements of key properties, such as overpotential, exchange current density, and Tafel slopes. A lack of precise and accurate data can lead to incorrect predictions and unreliable models. Accurate datasets with precise measurements of key properties can aid in the development of reliable machine learning models and enable the discovery of novel electrocatalysts with improved HER performance.
2. With the emergence of intelligent robots and 3D printing technology, the development of electrocatalysts has rapidly advanced. In fact, it has become possible for robots to predict electrocatalysts in their self-driving laboratories. To further enhance research and development efficiency, an innovative robotic platform will be developed by integrating machine learning with AI chemists and high-throughput experiments. This platform will aim to automatically optimize experimental design and improve research efficiency. It will also incorporate data-driven robotic synthesis, robot-assisted controllable preparation, and HER performance-oriented inverse design to effectively enhance the development of electrocatalysts.
3. Aside from the current effective descriptors, low-cost computation, and environmentally-friendly preparation techniques used for the HER, it is essential to establish more powerful multi-objective optimization models. These models are crucial in screening and predicting suitable electrocatalysts for HER, and will greatly improve the efficiency and accuracy of the screening process. By incorporating these models, the development of HER electrocatalysts can reach new heights, resulting in a more sustainable and efficient energy production.
4. The electrocatalytic activity of HER is mainly influenced by the electronic and geometrical structures of active sites at an atomic level. However, other macroscopic factors such as solvents and electrical fields can also impact the HER performance. To improve the predictability of novel electrocatalysts, it is essential to develop cross-scale models that incorporate experimental and environmental parameters. By doing so, the predictive power of electrocatalysts can be enhanced, leading to the development of more efficient HER electrocatalysts. Such advancements will facilitate the design and production of electrocatalysts that are better suited for HER, resulting in more sustainable and efficient energy production. However, incorporating experimental and environmental parameters into cross-scale models can dramatically increase the complexity of the modeling framework. Such an increase in complexity necessitates the employment of more advanced algorithms and substantial computational resources. Consequently, this can lead to a considerable escalation in both computational cost and the time required for simulations.

In summary, machine learning is a versatile and comprehensive quantitative methodology that holds significant potential for advancing research in the field of HER. Its adaptable and flexible nature makes it a promising tool for further progress in this domain. This review aims to inspire theoretical and experimental investigations into the use of machine learning, ultimately driving the field forward. Nevertheless, we emphasize the crucial role of rigorous scientific inquiry in unlocking the full potential of this approach. Therefore, this review aims to facilitate the widespread deployment of machine learning in high-performance HER electrocatalysts.
